# The Aerial Parts of *Agrimonia procera* Wallr. and *Agrimonia eupatoria* L. as a Source of Polyphenols, and Especially Agrimoniin and Flavonoids

**DOI:** 10.3390/molecules26247706

**Published:** 2021-12-20

**Authors:** Elżbieta Karlińska, Beata Romanowska, Monika Kosmala

**Affiliations:** 1 Institute of Food Technology and Analysis, Faculty of Biotechnology and Food Sciences, Lodz University of Technology, Stefanowskiego 2/22, 90-537 Łódź, Poland; monika.kosmala@p.lodz.pl; 2 Medicinal Plant Garden, Department of Pharmacognosy, Faculty of Pharmacy, Medical University of Lodz, Muszyńskiego 1, 90-151 Łódź, Poland; beata.romanowska@umed.lodz.pl

**Keywords:** *Agrimonia procera* Wallr., *Agrimonia eupatoria* L., *Rosaceae*, fragrant agrimony, common agrimony, polyphenols, ellagitannins, flavones, flavonols, agrimoniin, HPLC-DAD-MS

## Abstract

Plants of the genus *Agrimonia* L. perfectly fit the current trends in nutrition and food technology, namely, the need for raw materials with a high content of bioactive natural compounds, including polyphenols, which could be added to food. The composition of polyphenolics, including agrimoniin and flavonoids, in the aerial parts of *Agrimonia procera* Wallr. (*A. procera*) and *Agrimonia eupatoria* L. (*A. eupatoria*) (*Rosaceae*) was determined using HPLC-DAD-MS. The polyphenolic content of *A. procera* was found to be 3.9%, 3.2%, 2.9%, 1.8% and 1.1%, and that of *A. eupatoria* was determined to be 1.3%, 0.3%, 0.9%, 0.6% and 0.5% in the dry matter of leaves, stems, fruits, seeds and hypanthia, respectively. Except for *A. procera* hypanthia, agrimoniin was the main polyphenolic compound in the aerial parts of the studied *Agrimonia* species. Both plants are also a valuable source of flavonoid glycosides, especially apigenin, luteolin and quercetin. The obtained data indicate that both *A. procera* and *A. eupatoria* are potentially good sources of polyphenols (albeit significantly different in terms of their qualitative and quantitative composition), and may not only be a medicinal raw material, but also a valuable material for food use such as nutraceuticals or functional food ingredients.

## 1. Introduction

Discovering new health properties of plant-based foods is an increasingly important area of scientific endeavor. In light of recent nutritional and medical research, plant metabolites, and especially polyphenols, are responsible for the health-beneficial properties of many edible and medicinal plants. In particular, two groups of polyphenols, i.e., ellagitannins and flavonoids, have been the subject of extensive in vitro and in vivo studies [1,2,3,4,5].

Plants of the genus *Agrimonia* L., including the common agrimony *Agrimonia eupatoria* L. (*A. eupatoria*) and the fragrant agrimony *Agrimonia procera* Wallr. (*A. procera*) (synonym *Agrimonia odorata* Mill.), are sources of both ellagitannins and flavonoids. So far, plants of the genus *Agrimonia* L. have been primarily used for medicinal purposes, mainly in the form of infusions and decoctions [2,6,7,8]. In the case of agrimony, the medicinal raw material is the herb, i.e., the aerial parts of plants harvested during the flowering period, which occurs from early July to mid-August. The fruits (achenes) become mature soon after flowering [9]. In practice, this means that in the flowering period, clusters containing both flowers and developing or ripening agrimony fruits are found in addition to leaves and stems. Plants of the genus *Agrimonia* are resistant to diseases and a periodical lack of water, and do not require the use of plant protection products [9,10,11]. This makes them safe organic materials and provides an additional argument for expanding their nutritional use. They are characterized by a high yield of biomass and contain biologically active substances that affect human metabolism. Therefore, they perfectly fit the current trends in nutrition and food technology, namely, the need for raw materials with a high content of bioactive natural compounds, including polyphenols, which could be added to food.

In particular, two groups of polyphenols, i.e., ellagitannins and flavonoids abundant in these plants, have been the subject of extensive in vitro and in vivo studies [1,2,3,4,5]. It is therefore interesting to examine new practical perspectives for the use of plants of the genus *Agrimonia* L. in the food industry as a rich source of phytocomponents, including polyphenols.

The polyphenols contained in these species are ellagitannins (mainly agrimoniin) and flavonoids, including quercetin, kaempferol, apigenin, and luteolin [7,8,12,13,14,15]. Ellagitannins are hydrolyzable tannins—esters of 3,4,5,3’,4’,5’-hexahydroxydiphenoyl acid (HHDP), mostly with β-d-glucose. These polyphenols are characterized by a highly variable structure of their units due to the many ways of linking HHDP and sugar moieties, and by a tendency to form oligomers [16,17]. The health promoting properties of ellagitannins are related to their structure and the ability to successively release ellagic acid moieties from their molecules, which further degrade into urolithins in human and animal organisms [4,5]. Ellagitannins, ellagic acid, and their metabolites possess many biological properties with potentially beneficial effects on human health, such as inhibiting the development of civilization diseases, including cancer [18,19]. Agrimoniin, the main ellagitannin of *Agrimonia* species, is reported to have antibacterial, anti-inflammatory, and antioxidant properties [20,21]. It inhibits the development and proliferation of neoplastic cells and stimulates their apoptosis [22].

Flavonoids are derivatives of 2-phenyl-benzo-γ-pyrone with a C6-C3-C6 carbon skeleton formed by two benzene rings connected by a pyrone ring. Based on their structure, flavonoids are divided into six groups (flavanones; flavanols; flavones, including apigenin and luteolin; isoflavones; flavonols, including quercetin and kaempferol; and anthocyanins). The diverse biological activities of these compounds are attributable to the great variety of their chemical structures and the presence of different groups and moieties in flavonoid molecules, which determine their effects on cellular metabolism [23]. The flavonoids found in *Agrimonia* species have potent biological properties. Luteolin inhibits cholesterol biosynthesis, reduces low-density lipoproteins, and has a chemotherapeutic effect limiting the proliferation of certain types of cancer cells [24,25]. Apigenin accelerates the formation of nerve cells and strengthens neural connections in the brain, enhancing memory and learning functions [26]. *C*-glycoside derivatives of apigenin show vasodilating properties, improving circulation and heart muscle function [27]. Quercetin has a strong antioxidant effect, reduces the risk of cardiovascular diseases, and is also an anti-carcinogenic factor [28,29]. Kaempferol is a natural compound in cancer chemoprevention and treatment, and exhibits antioxidant and anti-inflammatory activity, e.g., it prevents neuronal damage in inflammatory conditions such as Alzheimer’s and Parkinson’s diseases [30,31].

The aim of this study was to investigate the composition and quantitative content of ellagitannins and flavonoids in the aerial parts of *A. procera* and *A. eupatoria* in the main stages of plant development. To the best of the authors’ knowledge, the polyphenolic composition of individual aerial parts of the two species of agrimony has not been described in the literature to date. Another aim of this study was to gain new knowledge about the distribution of the studied polyphenols in those parts of the two *Agrimonia* species which are used as medicinal raw materials and may also be applied as food ingredients.

## 2. Results and Discussion

The polyphenols found in the study material were identified, and their qualitative and quantitative composition in the main aerial parts of agrimony was determined by stage of plant development.

### 2.1. Tentative Identification of Major Polyphenols in the Arial Parts of Common Agrimony and Fragrant Agrimony

Table 1 presents data on the retention times, UV-vis data, molecular weight (MW), and fragmentation ions (MS/MS spectrum) of the polyphenols identified in *A. procera* and *A. eupatoria* leaves, stems, fruits, seeds, and hypanthia, and Figure S1 in Supplementary Materials contains the chromatograms recorded for the leaves of both species. Among the identified polyphenols are ellagitannins, phenolic acids, and flavonoids, including flavonols and flavones. The main polyphenolic compound in agrimony is agrimoniin (MW 1870), named after the Latin term for the plant. Isolated for the first time in 1982 from the hairy agrimony, agrimoniin is the first known ellagitannin with a dimeric structure [26]. Agrimoniin, ellagic acid (MW 302), and KpCG (kaempferol-3-*O*-β-d-(6″-*E*-*p*-coumaroyl)-glucopyranoside, tiliroside, MW 594) were present in all analyzed morphological parts of both *A. eupatoria* and *A. procera*, in all stages of their development. The data in Table 1 also show that the occurrence of luteolin, apigenin, quercetin and kaempferol glycosides varied depending on the species and morphological part of the plant. Compounds tentatively identified as quercetin arabinoglycoside (MW 596) and kaempferol 3-*O*-glucoside (MW 448) were only present in *A. procera* (in all aerial parts except for the latter compound, which was absent from the seeds). Quercetin arabinoglycoside was identified in *A. procera* for the first time in this study, and according to the mass spectra, this compound could be attributed either to arabinoglucoside or arabinogalactoside. This is a relatively rare compound with a structure similar to quercetin 3-*O*-rhamnoglucoside. Quercetin 3-*O*-arabinoglucoside was previously identified in *Ruprechtia salicifolia* leaves [3] and *Mangifera indica* L. fruit peel [32].

In turn, apigenin 8-*C*-glucoside (vitexin, MW 432), its isomer apigenin-6-*C*-glucoside (isovitexin, MW 432), and quercetin 3-*O*-rhamnoside (quercitrin, MW 448) were present only in *A. eupatoria*, in all aerial parts except seeds. Apigenin 7-*O*-glucuronide (MW 446) occurred in all aerial parts of *A. eupatoria*, but only in the seeds of *A. procera*. The presence of apigenin 8-*C*-glucoside and apigenin 6-*C*-glucoside has been recognized in the literature as being characteristic of *A. eupatoria*, important in distinguishing materials derived from *A. eupatoria* and *A. procera* [8]. Quercetin 3-*O*-rhamnoglucoside (rutin, MW 610), quercetin 3-*O*-galactoside (hyperoside, MW 464), luteolin 7-*O*-glucuronide (MW 462), and apigenin 7-*O*-glucoside (MW 432) were found in all aerial parts of both species, with the exception of seeds. In addition to agrimoniin, ellagic acid and KpCG, the seeds of *A. procera* and *A. eupatoria* contained quercetin arabinoglycoside and apigenin 7-*O*-glucuronide, respectively.

There are several publications describing the identification of polyphenolic compounds in the dried herb of *A. eupatoria* [7,12,13,14,15,34], as well as the herb of both studied agrimony species [8]. The present data are largely consistent with the results of the aforementioned studies, concerning mainly dried herb, but as stated before, our work describes for the first time a compound with MW 596 in *A. procera*, which is tentatively identified as quercetin arabinoglycoside. This compound has not been previously described in the literature as occurring in this plant.

### 2.2. The Content and Percentage Shares of Polyphenols in the Aerial Parts of Agrimonia Plants in the Main Stages of Plant Development

The content of ellagitannins and flavonoids (mg/100 g dry weight (DW)) and their percentage shares in total polyphenols in the main morphological parts (leaves, stems, fruits, seeds, and hypanthia) of *A. procera* and *A. eupatoria* in the five main stages of plant development are given in Table 2 and Table 3, respectively. The content of polyphenols in both plant species was analyzed during the development of vegetative parts and the appearance of inflorescence (S1), during the flowering period (S2), during the development of fruits (achenes) and seeds (S3), at the beginning of fruit and seed ripening (S4), and at full maturity of fruits and seeds (S5) [35].

#### 2.2.1. *Agrimonia procera* Wallr. Composition

In the analyzed period of plant development, the mean contents of total polyphenols, in mg/100 g DW, in *A. procera* leaves, stems, fruits, seeds, and hypanthia were 3963.1 ± 53.1, 3233.8 ± 669.4, 2908.1 ± 499.5, 1809.1 ± 379.2, and 1109.1 ± 374.9, respectively, with those of agrimoniin being 1855.6 ± 342.8, 3038.8 ± 628.5, 1676.5 ± 321.8, 1660.1 ± 388.4, and 339.0 ± 150.3, respectively. When analyzing the quantitative composition of polyphenols in individual stages, it can be seen that the content of agrimoniin in *A. procera* leaves was significantly higher in S1 and S2 as compared to the other stages. In *A. procera* stems, the amount of this ellagitannin was the greatest in S5, with its content in S3 and S4 being significantly higher than in S1 and S2. In *A. procera* fruits and seeds, there was significantly more agrimoniin in S3 than in S4 and S5, unlike in hypanthia, in which its content in S3 and S4 was less than a half of that in S5.

The mean content of total luteolin glycosides, in mg/100 g DW, in *A. procera* leaves, stems, fruits, seeds, and hypanthia was 195.5 ± 22.0, 8.9 ± 2.8, 87.1 ± 11.5, 0, and 51.4 ± 0.5, respectively. In *A. procera* leaves, a significant decrease in the content of luteolin 7-*O*-glucuronide in S5 relative to S1 was accompanied by an increase in luteolin 7-*O*-glucoside. In *A. procera* fruits, the content of luteolin 7-*O*-glucoside was constant throughout the analyzed stages of plant development, while that of luteolin 7-*O*-glucuronide remained the same in S3 and S4, being statistically higher than in S5. In *A. procera* hypanthia, luteolin glycoside levels were similar in S3 and S4, and increased in S5.

In the aerial parts of *A. procera*, the mean content of total apigenin glycosides, in mg/100 g DW, was 1053.8 ± 122.0, 14.8 ± 6.5, 135.7 ± 18.6, 0, and 56.8 ± 0.3 in the leaves, stems, fruits, seeds, and hypanthia, respectively. In *A. procera* leaves, apigenin 7-*O*-glucuronide levels in S1 and S2 were significantly higher than in the other stages, while those of apigenin 7-*O*-glucoside were relatively constant, except for S3. In *A. procera* fruits, a significantly higher content of apigenin 7-*O*-glucuronide was observed in S3 and S4, and that of apigenin 7-*O*-glucoside in S3, as compared to the remaining plant development stages.

The mean content of total quercetin glycosides in the analyzed *A. procera* development stages, in mg/100 g DW, was 769.8 ± 85.1, 124.6 ± 35.5, 750.0 ± 120.2, 4.2 ± 3.2, and 548.4 ± 138.9 in the leaves, stems, fruits, seeds, and hypanthia, respectively. In *A. procera* leaves, the content of quercetin arabinoglycoside and quercetin 3-*O*-rhamnoglucoside was the highest in S1 and S2, and that of quercetin 3-*O*-galactoside was highest in S5. In *A. procera* stems and hypanthia, quercetin glycosides were the most abundant in S5, and in fruits in S3.

In the analyzed stages of plant development, the mean content of kaempferol derivatives, in mg/100 DW, was 126.7 ± 15.8, 10.8 ± 1.6, 52.1 ± 9.7, 4.6 ± 0.4, and 27.3 ± 14.8, in *A. procera* leaves, stems, fruits, seeds and hypanthia, respectively. In *A. procera* leaves, the amount of kaempferol 3-*O*-glucoside was similar in S1 and S2, and that of KpCG remained at a relatively constant level between S1 and S3. In the fruits, kaempferol 3-*O*-glucoside was the most abundant in S3, and KpCG in S3 and S5, with the differences being statistically significant as compared to the other stages.

#### 2.2.2. *Agrimonia eupatoria* L. Composition

The data presented in Table 3 show that in the analyzed period of *A. eupatoria* development, the mean content of polyphenols, in mg/100 g DW, was 1316.5 ± 130.6, 302.3 ± 95.2, 946.5 ± 283.3, 583.8 ± 193.2, and 546.9 ± 190.9 in the leaves, stems, fruits, seeds and hypanthia, respectively, with the content of agrimoniin being 426.7 ± 66.8, 226.8 ± 91.2, 704.8 ± 248.0, 529.9 ± 189.2, and 277.8 ± 104.2, respectively. *A. eupatoria* leaves revealed a specific pattern of change in agrimoniin concentration: it was similar in S1 and S2, and then it increased significantly in S3 only to decrease over the next two stages to the initial levels. In *A. eupatoria* stems, agrimoniin was the most abundant in S4, with its levels in S1 and S2 being similar and significantly lower than in S3 and S5. In *A. eupatoria* fruits and seeds, the highest content of agrimoniin was recorded in S1, declining significantly over the next two stages. The opposite tendency was found in *A. eupatoria* hypanthia, where agrimoniin concentration was significantly higher in S5 than in S3 and S4.

The mean content of total luteolin glycosides, in mg/100 g DW, in *A. eupatoria* leaves, stems, fruits, seeds, and hypanthia was 135.8 ± 25.4, 7.3 ± 3.0, 3.4 ± 0.6, 0, and 3.6 ± 2.1, respectively. In *A. eupatoria* leaves, the highest level of luteolin 7-*O*-glucuronide was found in S1 and S2, and that of luteolin 7-*O*-glucoside in S5.

The content of total apigenin glycosides in *A. eupatoria* leaves, stems, fruits, seeds, and hypanthia was 465.2 ± 50.6, 18.1 ± 3.1, 128.6 ± 21.4, 2.6 ± 0.4, and 161.8 ± 42.3, respectively. In *A. eupatoria* leaves, apigenin 7-*O*-glucoside was the most abundant in S5, while apigenin 7-*O*-glucuronide reached the lowest levels in S3 and S4, apigenin 8-*C*-glucoside in S4, and apigenin-6-*C*-glucoside in S3; in the remaining stages of plant development, the concentrations of those compounds were relatively constant. The levels of the analyzed apigenin derivatives in *A. eupatoria* fruits and seeds were significantly higher in S3 than in the other two stages. In the hypanthia, changes in the content of *C*-glycoside derivatives of apigenin and apigenin 7-*O*-glucuronide throughout the analyzed plant development stages were not statistically significant, while apigenin 7-*O*-glucoside levels were significantly higher in S4 and S5 as compared to S3.

The mean content of total quercetin glycosides in the analyzed period of *A. eupatoria* development, in mg/100 g DW, was 237.9 ± 74.9, 27.5 ± 13.7, 35.8 ± 7.7, 0, and 50.5 ± 24.4 in the leaves, stems, fruits, seeds, and hypanthia, respectively. In *A. eupatoria* leaves, quercetin 3-*O*-rhamnoglucoside and quercetin 3-*O*-galactoside were the most abundant in S5, and quercetin 3-*O*-rhamnoside in S1 and S2. In *A. eupatoria* stems, glycosidic derivatives of quercetin were found at the highest concentration in S5, while in the fruits, their amount in S3 was significantly greater than in S4 and S5. In *A. eupatoria* hypanthia, the amount of quercetin 3-*O*-rhamnoglucoside was significantly higher in S4 and S5, and that of quercetin 3-*O*-rhamnoside in S5, as compared to the other plant development stages, while quercetin 3-*O*-galactoside levels in the hypanthia were relatively constant throughout the *A. eupatoria* development stages.

The mean content of KpCG in the analyzed *A. eupatoria* development stages, in mg/100 g DW, was 42.4 ± 106, 8.9 ± 2.2, 15.9 ± 3.7, 2.2 ± 0.6, and 11.6 ± 4.3, in the leaves, stems, fruits, seeds, and hypanthia, respectively. In *A. eupatoria* leaves, a significantly higher content of KpCG was observed in S1 and S2 as compared to the other stages. In *A. eupatoria* fruits, KpCG was most abundant in S1, and in the hypanthia in S5, with the differences being statistically significant with respect to the other stages.

The current findings regarding the content of agrimoniin in the dry matter of *A. eupatoria* leaves (4.3 mg/g DW) as well as their flavonoid composition (1.0 mg quercetin 3-*O*-galactoside, 1.4 mg luteolin 7-*O*-glucuronide, and 4.6 mg apigenin 7-*O*-glucuronide per g DW) are consistent with the results of commercial herb trials [13]. Results for four samples of *A. eupatoria* aerial parts revealed the following ranges for these compounds, in mg/g DW: 2.63–5.39 for agrimoniin, 1.18–1.72 for quercetin 3-*O*-galactoside, 1.48–2.33 for luteolin 7-*O*-glucuronide, and 1.67–2.28 for apigenin 7-*O*-glucuronide [13].

Granica et al. [8] compared herbal samples, including commercial preparations, of the two studied agrimony species in terms of agrimoniin content. Depending on plant provenance, the content of agrimoniin in three samples of *A. procera* herb determined by HPLC was from 21.31 to 37.05 mg/g DW, with a mean of 28.4 mg/g DW, while its content in six samples of *A. eupatoria* herb was significantly lower, ranging from 1.22 to 6.77 mg/g DW, with a mean of 3.3 mg/g DW. In this paper, agrimoniin concentrations in the leaves and stems in the harvest stage (at the beginning of flowering) were 20.0 and 27.0 mg/g DW for *A. procera*, and 4.2 and 2.3 mg/g DW for *A. eupatoria*, respectively, which is consistent with the publication cited above. It is worth noting, however, that agrimoniin content in *A. procera* leaves was lower than in the stems, while the opposite was true of *A. eupatoria*. Thus, agrimoniin concentration in commercial herbs is determined not only by plant species, but also by the relative proportions of leaves and stems.

Gräber et al. [36] reported that powdered *A. procera* leaves and combined powdered *A. procera* leaves and stems contained 12.7 and 6.38 mg of agrimoniin per g DW, respectively. However, in that case, agrimoniin was determined in extracts obtained with hot deionized water, which does not guarantee its complete extraction from the plant material. For this reason, the aforementioned figures are substantially lower than the present findings.

The obtained data indicate that the content of flavonoids in the studied agrimony species was high as compared to other plants that are considered good sources of these compounds. For example, in artichoke, the content of apigenin 7-*O*-glucuronide and luteolin 7-*O*-glucuronide has been reported to be 0.49 and 0.13 mg/g DW, respectively [37], while in the present study, *A. procera* and *A. eupatoria* leaves contained 9.39 and 2.06 mg of apigenin 7-*O*-glucuronide per g DW, and 1.61 and 1.19 mg of luteolin 7-*O*-glucuronide per g DW, respectively. In mung beans (*Vigna radiata* L.), the concentration of apigenin 8-*C*-glucoside and apigenin 6-*C*-glucoside is in the range of 8.1–11.9 and 8.6–12.5 mg/g DW, respectively [38]. The leaves and flowers of different hawthorn species (*Crataegus* spp.) have 0.20–5.51 and 0.01–2.00 mg of apigenin 8-*C*-glucoside per g DW, respectively [1], while the flowers and leaves of 15 hawthorn species have been reported to contain from 0.51 to 3.90 mg of apigenin 8-*C*-glucoside per g DW [39]. For comparison, in the present investigation, the content of apigenin 8-*C*-glucoside and apigenin 6-*C*-glucoside in *A. eupatoria* leaves was 0.50 and 1.50 mg/g DW, respectively. In a study by Rusalepp et al. [40], *Hypericum perforatum* from two harvest seasons had 17–27 mg of quercetin 3-*O*-galactoside per g DW, as compared to ca. 1 mg/g DW in the leaves of *A. procera* and *A. eupatoria* in this paper.

### 2.3. Differences in Polyphenol Content between the Studied Morphological Parts of Agrimonia Species

The data collected in Table 2 and Table 3 were used to determine the concentrations and sites of accumulation of individual polyphenols in the aerial parts of *A. procera* and *A. eupatoria*. The data on polyphenol concentration in the leaves and stems of the two species are of significant practical importance, as they help to explain substantial discrepancies in the assessment of the composition of dried agrimony herb as a commercial raw material consisting of varying proportions of leaves and stems harvested in different growth stages. In addition, the potential use of *Agrimonia* species as a source of polyphenols, and especially ellagitannins and flavonoids, requires knowledge of the content of individual compounds in the various aerial parts and the most favorable stage of plant development for harvesting and obtaining the polyphenols.

The presented data show that, with the exception of *A. procera* hypanthia, agrimoniin was the main polyphenolic compound in the aerial parts of the studied agrimony species. The mean share of agrimoniin in the pool of determined polyphenols was almost 95%, 50%, 60%, and 90%, respectively, in *A. procera* stems, leaves, fruits and seeds in the analyzed period of plant development. Among the analyzed *A. procera* aerial parts, the hypanthia had the lowest concentration of agrimoniin, on average accounting for only 30% of the total polyphenols in that morphological part.

It is noteworthy that *A. eupatoria* differs considerably from *A. procera* in its characteristics of agrimoniin accumulation and distribution among aerial parts. Throughout the entire analyzed period of plant development, the highest concentration of agrimoniin in *A. eupatoria* was found in its fruits and seeds, on average accounting for almost 75% and 90% of total polyphenols, respectively. The parts with the lowest agrimoniin concentrations were *A. procera* stems and hypanthia, but the share of agrimoniin in the pool of polyphenols found in these morphological parts was relatively high (75% and 50.8%, respectively).

When determining agrimoniin content in *Agrimony* species, attention should be paid to differences in the ellagic acid share in total ellagitannins between individual morphological parts. Ellagic acid is a polyphenolic compound whose presence in plant tissues may result from both ellagitannin hydrolysis and the natural metabolic processes associated with plant growth and development. The hydrolysis of ellagitannins may be triggered by physical (e.g., light) and chemical (presence of acids and bases) factors, as well as by the action of enzymes, leading to the release of 3,4,5,3’,4’,5’-hexahydroxydiphenic acid (HHDP) molecules, which undergo spontaneous lactonization to ellagic acid, increasing its concentration in the analyzed material. The share of ellagic acid in total polyphenols in individual aerial parts of the studied agrimony species was also relatively low throughout plant development: 0.6% and 1% in *A. procera* and *A. eupatoria* leaves, and 1% and 4.6% in *A. procera* and *A. eupatoria* stems, respectively. The shares of ellagic acid in total polyphenols in the fruits, seeds and hypanthia of the studied agrimony species were higher, but they still did not exceed 10%.

Plants of the genus *Agrimonia* are a valuable source of flavonoids, including apigenin, luteolin, quercetin, and kaempferol derivatives. Flavones, i.e., apigenin and luteolin glycosides, tended to accumulate in the leaves of the studied agrimony species. On average, throughout plant growth, combined flavone glycosides accounted for over 30% of total leaf polyphenols, both in *A. procera* and *A. eupatoria*. The aerial parts with the highest concentrations of quercetin and kaempferol derivatives were the leaves and fruits in the case of *A. procera* as compared to the leaves and, to a lesser extent, fruits and hypanthia, in the case of *A. eupatoria*. Throughout the analyzed period of *A. procera* development, flavonol glycosides on average accounted for nearly 22.6%, 27.6%, and 51.4% of total polyphenols in the leaves, fruits, and hypanthia of that species, respectively. In *A. eupatoria*, the share of glycoside flavonol derivatives in the pool of polyphenols in the leaves, fruits and hypanthia was 23.2%, 5.6%, and 11.0%, respectively.

The only flavonoids found in the seeds were quercetin arabinoglycoside and KpCG (in *A. procera*), and apigenin 7-*O*-glucuronide and KpCG (in *A. eupatoria*), which means that this morphological plant part is neither a site of synthesis nor accumulation of these compounds. The dominant polyphenolic component in the seeds was agrimoniin, whose average share in the total polyphenols was over 90% both in *A. procera* and *A. eupatoria.*

The presented data indicate that the content of individual polyphenols in dried agrimony herb, including commercially available preparations, will largely depend on the relative shares of the various plant parts. A higher proportion of *A. procera* stems will significantly increase the content of agrimoniin while reducing that of flavonoids. As regards the aerial parts of *A. eupatoria*, a greater proportion of the stems will lead to a decreased content of agrimoniin and flavonoids. These relationships explain the substantial discrepancies in the quantitative polyphenolic composition of commercial agrimony preparations observed in the literature. The presented data also suggest the possibility of nutritional use of the studied agrimony species, and especially their leaves and stems as rich sources of polyphenols. In particular, *A. procera* leaves are a promising potential raw material because in S1 they accumulate large amounts of agrimoniin, apigenin derivatives, and luteolin in the form of bioavailable glucuronides and quercetin glycosides. On the other hand, valuable *C*-glycoside derivatives of apigenin are present in *A. eupatoria* leaves, which also contain a moderate amount of agrimoniin. S1, when the vegetative plant parts develop, seems to be particularly suitable for harvesting *A. procera* and *A. eupatoria* for nutritional use, especially as regards the leaves, which not only contain desirable compounds, but are also soft and juicy in that stage.

The stems and fruits, especially of *A. procera*, are rich in agrimoniin, particularly in the final stages of generative development (S4 and S5), and so they can be considered a good material for obtaining this ellagitannin. The nutritional use of *Agrimonia* plants would complement the existing dietary sources of polyphenols (fruits and vegetables) and promote their application in various forms as medicinal plants. Due to the high content of polyphenols, both plant species could be used as potential nutraceuticals or functional food ingredients.

In the literature, there are few data on the variability of polyphenolic composition in the vegetative and/or generative stages of other plants belonging to the family *Rosaceae*, including strawberries. Gasperotti et al. [41] showed that in the fruits of six varieties of strawberries (*Fragaria pineanasa* Duch.), the content of agrimoniin tends to decrease in successive stages of fruit development. Depending on the variety, strawberry fruits contain agrimoniin, in mg/kg FW, in quantity: green fruits—132.0–419.6; veraison fruits—132.7–286.7; ripe fruits—60.6–190.8; and overripe fruits—38.1–139.9. The above plants of the genus *Agrimonia*, and especially *A. procera*, can be considered a valuable source of diet ellagitannins.

In a previous publication [42], the authors studied the qualitative and quantitative composition of ellagitannins characteristic of *Fragaria* × *ananassa* in the roots, leaves, and fruits of six dessert strawberry cultivars in the main stages of plant development. Analysis of changes in the content of ellagitannins, including agrimoniin, in the vegetative and generative growth stages revealed a decreasing tendency over time, with the decline being dependent on the morphological part of the plant and strawberry cultivar.

### 2.4. PCA Analysis of the Polyphenolic Profiles of A. procera and A. eupatoria

**Figure 1:**
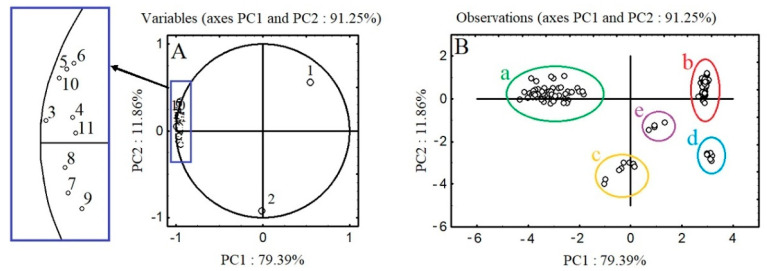
Principal component analysis (PC1 and PC2) of *A. procera* at five development stages. (**A**): The loading values of response values (polyphenols concentration) on PC1 and PC2 from PCA. (**B**): Observations on the same PCA axes. Variables: 1—agrimoniin; 2—ellagic acid; 3—luteolin 7-*O*-glucuronide; 4—luteolin 7-*O*-glucoside; 5—apigenin 7-*O*-glucuronide; 6—apigenin 7-*O*-glucoside; 7—quercetin arabinoglycoside; 8—quercetin 3-*O*-rhamnoglucoside; 9—quercetin 3-*O*-galactoside; 10—kaempferol 3-*O* glucoside; 11—sum of kaempferol-3-*O*-β-d-(6″-E-p-coumaroyl)-glucopyranoside. Observations: cluster a: leaves of *A. procera* in all analyzed stages of development; cluster b: stems of *A. procera* in all analyzed stages of development; cluster c: fruits of *A. procera*: during the development of fruits and seeds (S3), at the beginning of fruit and seed ripening (S4), and at full maturity of fruits and seeds (S5); hypanthia in full maturity of fruits and seeds (S5); cluster d: seeds of *A. procera*: during the development of fruits and seeds (S3), at the beginning of fruit and seed ripening (S4), and at full maturity of fruits and seeds (S5); cluster e: hypanthia of *A. procera*: during the development of fruits and seeds (S3), and at the beginning of fruit and seed ripening (S4).

**Figure 2:**
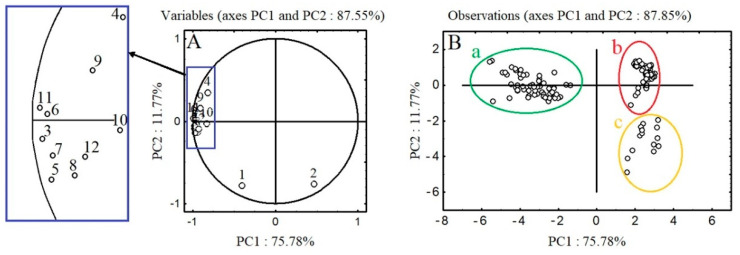
Principal component analysis (PC1 and PC2) of *A. eupatoria* at five development stages. (**A**) The loading values of response values (polyphenols concentration) on PC1 and PC2 from PCA. (**B**) Observations on the same PCA axes. Variables: 1—agrimoniin; 2—ellagic acid; 3—luteolin 7-*O*-glucuronide; 4—luteolin 7-*O*-glucoside; 5—apigenin 7-*O*-glucuronide; 6—apigenin 7-*O*-glucoside; 7—apigenin 8-*C*-glucoside; 8—apigenin 6-*C*-glucoside 9—quercetin 3-*O*-rhamnoglucoside; 10—quercetin 3-*O*-galactoside; 11—quercetin 3-*O*-rhamnoside; 12—sum of kaempferol-3-*O*-β-d-(6″-E-p-coumaroyl)-glucopyranoside. Observations: cluster a: leaves of *A. eupatoria* in all analyzed stages of development; cluster b: stems of *A. eupatoria* in all analyzed stages of development; hypanthia of *A. eupatoria* during the development of fruits and seeds (S3), and at the beginning of fruit and seed ripening (S4); cluster c: fruits and seeds of *A. eupatoria* in all analyzed stages of development; hypanthia of *A. eupatoria* during full maturity of fruits and seeds (S5).

PCA analysis was used to visualize potential grouping in the dataset consisting of the polyphenolic profiles of the aerial parts (leaves, stems, fruits, seeds, and hypanthia) of *A. procera* and *A. eupatoria* in the main stages of plant development. The contribution of each variable to the first two factors and the planar distribution of individual samples for *A. procera* and *A. eupatoria* are shown in Figure 1 and Figure 2, as well as Tables S1 and S2 in Supplementary Materials. The presented data show that in the case of both analyzed agrimony species, the first two main factors (PC1 and PC2) with eigenvalues higher than one explain 91.25% and 87.55% of the variance in data concerning *A. procera* and *A. eupatoria*, respectively.

Analysis of the obtained relationships shows that the first axis (PC1) explains 79.39% and 75.78% of the variance in *A. procera* and *A. eupatoria*, respectively; the eigenvalues of 8.73 and 9.09, respectively, indicate that it represents information originally explained by nine variables (individual polyphenolic compounds). The second factor (PC2) accounts for 11.86% and 11.77% of the total variance in *A. procera* and *A. eupatoria* data, respectively.

PCA plots based on polyphenolic profiles reveal distinct differentiation between the various aerial parts of *A. procera* and *A. eupatoria* (Figure 1 and Figure 2). In the case of *A. procera*, five clearly defined clusters are distinguished: clusters *a*, *b* and *c* are formed by *A. procera* leaves, stems, and seeds in all stages of development, respectively, cluster *d* corresponds to the fruits in S3–S5 and the hypanthia in S5, and cluster *e* contains the hypanthia in S3 and S4. These distinctions are mainly due to PC1.

*A. procera* leaves form a compact cluster (*a* in Figure 1) defined by the content of individual flavonoids and agrimoniin, the stems (cluster *b*) are characterized by high agrimoniin content, and the seeds (*c*) are defined by high agrimoniin and ellagic acid concentrations combined with low flavonoid levels. The fruits in S3–S5 and the hypanthia in S5 (cluster *d* in Figure 1) show a clear similarity in quantitative polyphenolic composition, mainly in terms of ellagic acid and flavonoids. The hypanthia in S3 and S4 (cluster *e*) are located between the leaves, stems, and seeds, being in closer proximity to the latter two categories of plant parts.

As regards *A. eupatoria*, PCA analysis showed smaller variation in the analyzed data. Figure 2 shows three distinct clusters: cluster *a* is formed by the leaves in all periods of plant development; cluster *b* consists of the stems in all growth stages and the hypanthia in S3 and S4; and cluster *c* is formed by the fruits and seeds in S3–S5 and the hypanthia in S5. *A. eupatoria* leaves form a cluster clearly separated from the other two, primarily characterized by high flavonoid concentrations, and to a lesser extent by the content of agrimoniin. The location of the stems as well as the hypanthia in S3 and S4 in cluster *b* results from similar levels of agrimoniin, quercetin derivatives, luteolin 7-*O*-glucuronide, apigenin 7-*O*-glucoside, and KpCG in these samples, while the assignment of the hypanthia in S5 to cluster *c* results from their affinity to the fruits and seeds in terms of agrimoniin and ellagic acid content. The close proximity of clusters *b* and *c* on the PCA plot is due to the similarity between *A. eupatoria* stems, fruits, seeds, and hypanthia, mainly due to low flavonoid content in these plant parts.

## 3. Materials and Methods

### 3.1. Plant Material

The raw material was obtained from the Medicinal Plants Garden (MPG) of the Department of Pharmacognosy, Faculty of Pharmacy, Medical University of Lodz, Łódź, Poland) (coordinates 51.44° N, 19.24° E, at 187 m above sea level). The authenticity of the plant material was confirmed: (1) by the MPG based on the morphological features of *Agrimonia procera* Wallr. and *Agrimonia eupatoria* L. (*Rosaceae*) using literature sources [9,42]; (2) by the Institute of Horticulture in Skierniewice, Department of Applied Biology based on the number of *A. procera* and *A. eupatoria* chromosomes (analysis by Małgorzata Podwyszyńska, D.Sc. and Agnieszka Marasek-Ciołkowska, D.Sc.); and based on the declaration of the Botanical Garden in Lodz on the delivery of *A. eupatoria* seed material. The voucher specimens 20180613/ARPR/MPG (for *A. procera* Wallr.) and 20180613/AREU/MPG (for *A. eupatoria* L.) are deposited in MPG.

### 3.2. Course of the Experiment

#### 3.2.1. Production of Seedlings

The seed material for *A. procera* cultivation was from the MPG and that for *A. eupatoria* from the Botanical Garden in Lodz (Łódź, Poland) (in both cases collected in 2015). The achenes were immersed in water for 8 days, and then spot-sown in leaf compost from the MPG at a depth of ca. 0.5 cm, on concrete tables in a greenhouse. During the production of seedlings, tending procedures consisted of irrigation and the removal of other plants germinating in the substrate.

#### 3.2.2. Cultivation, Fertilization and Tending Treatments during the Growing Season

A micro-plantation for producing raw material for laboratory tests was established at the MPG in the spring of 2016. Before planting, sifted leaf compost, produced on site, was spread over selected plots in an ca. 4 cm-thick layer and mixed with the topsoil. *A. procera* and *A. eupatoria* seedlings were planted into that substrate after it hardened, in a grid of 0.25 × 0.30 m

In the following years, tending procedures during the growing season consisted of preventing the growth of other plants as well as drip irrigation to supplement natural rainfall. In the spring of 2017 and 2018, the plants were fertilized organically with screened compost produced on site.

#### 3.2.3. Harvesting, Drying and Storage of the Raw Material

*A. procera* and *A. eupatoria* were harvested in the third year of cultivation, i.e., in 2018, from June to September. For determination, seven plants of each *Agrimonia* species were randomly collected by hand at one time from the inner rows of the plots (omitting the edges). The plants were cut with secateurs about 15–20 cm above the ground. They were collected at selected stages of development, i.e., during the development of vegetative parts and the appearance of inflorescence (S1), during the flowering period (S2), during the development of fruits (achenes) and seeds (S3), at the beginning of fruit and seed ripening (S4), and at full maturity of fruits and seeds (S5) [35]. Samples were collected on five days for S1; nine days for S2; four days for S3 and S5; and seven days for S4, at 3–4-day intervals.

During S1, aerial parts of the plants were collected for analysis from the moment when harvestable vegetative plant parts reached 70% of the final size and inflorescence emerged with the first individual flowers visible (still closed) until the harvestable vegetative plant parts reached the final size and the first flower petals became visible. During this period, samples were collected on five dates: the 13th, 16th, 20th, 24th, and 28th of June. S2 extended from the beginning of flowering (about 10% of flowers open) through full flowering (at least 50% of flowers open, the first petals may be fallen) until the end of flowering (flowers fading), with the majority of petals fallen or dry. During S3, the plants collected for analysis contained developed fruits (35–45% of the final fruit size, fruit color green, seeds well-formed, large, white); at the top of the cluster, about 5–10% of the flowers were still in bloom. In S4, further development and enlargement of fruits and seeds was observed; about 50% of the fruits changed color from green to brown, and the seeds were cream-white, large, and hard. No blooming flowers were observed. In S5, nearly all fruits and seeds were ripe. The harvested plants were dried and spread between sheets of paper on tables in a shaded greenhouse in the MPG, at about 25–35 °C.

Subsequently, the plant material was transferred to the Institute of Technology and Food Analysis, Lodz University of Technology (Łódź, Poland), where the dried aerial parts collected at the various dates were divided into their morphological parts. The plant material collected in S1 and S2 was divided into stems and leaves, while the material collected in S3, S4, and S5 was divided into stems, leaves, and fruits. The leaves and stems of both tested agrimony species from each harvest date (biological replicates, BRs) were ground in an IKA A11 laboratory mill (Staufen, Germany). Due to their small mass share in the aerial parts, fruits were not analyzed at each harvest date as separate BRs, but were incorporated into average samples. In addition, about 60 fruits of each species of agrimony at a given stage of development were separated into seeds and hypanthia the day after harvest. The fruits were incised with a sharp instrument so as not to damage the seeds, and then the seeds were removed from the hypanthia. In S3, S4, and S5, the fruits (whole), seeds, and hypanthia of the two species of agrimony were ground in a mortar. The material was stored in sealed polypropylene containers at −18 °C until analysis.

### 3.3. Polyphenol Extraction from the Stems and Seeds of A. procera and A. eupatoria

Polyphenol extraction from *A. procera* and *A. eupatoria* stems and seeds was performed three times in a mixture of acetone/water/formic acid (70/29.9/0.1 *v/v/v*) (mixture A) according to the method described by Karlińska et al. [41], which is the most efficient way of extracting polyphenols from plant tissue containing ellagitannins and small amounts of flavonoids (unpublished studies on *Agrimonia* species). Approximately 350 mg of ground material was weighed into 7 mL polypropylene test tubes with stoppers, 4 mL of mixture A was added, mixed thoroughly using a vortex, and placed in an IS-4 ultrasonic bath (Intersonic, Olsztyn, Poland) for 15 min. After that time, the contents of the tubes were centrifuged in an MPW-260R laboratory centrifuge (Med Instruments, Warsaw, Poland) at 10,000× *g*. Subsequently, the supernatant was decanted from the pellet into 10 mL volumetric flasks. The residue remaining in the tube was subjected to two further extractions with 3 mL of mixture A in the same way. The resulting extracts were combined in a 10 mL volumetric flask and filled to the mark with mixture A. Extracts obtained with mixture A were diluted in methanol in a 1:5 (*v*/*v*) ratio, centrifuged in an MPW-260R laboratory centrifuge (Med Instruments, Warsaw, Poland) at 16,000× *g*, transferred to chromatographic vials, and subjected to HPLC analysis as described in Section 3.6 and Section 3.7. Extraction was performed in duplicate for each sample.

### 3.4. Polyphenol Extraction from the Leaves, Fruits, and Hypanthia of A. procera and A. eupatoria

Ellagitannin extraction from *A. procera* and *A. eupatoria* leaves, fruits, and hypanthia was performed successively: three times in a 70/29.9/0.1 (*v*/*v*/*v*) mixture of methanol/water/formic acid (mixture B), and then three times in mixture A, which is the most efficient way of extracting polyphenols from plant tissue containing both ellagitannins and flavonoids (unpublished studies on *Agrimonia* species). Extraction was performed as described in Section 3.4, except that the residue from the extraction with mixture B was subjected to an analogous extraction with mixture A. The extracts obtained with mixtures B and A were collected separately into two 10 mL volumetric flasks.

The extracts obtained with mixture B were diluted in methanol in a 1:4 (*v*/*v*) ratio, while those obtained with mixture A were diluted in methanol in a 1:2 (*v*/*v*) ratio. Subsequently, the diluted extracts were centrifuged in a MPW-260R laboratory centrifuge (Med Instruments, Warsaw, Poland) at 16,000× *g*, transferred to chromatographic vials and subjected to HPLC analysis as described in Section 3.6 and Section 3.7. Extraction was performed in duplicate for each sample.

### 3.5. Identification of Polyphenols

Identification of polyphenols was performed according to the methodology described in the previous publication [43] using a Dionex Ultimate 3000 HPLC coupled with a DAD and Q Exactive Orbitrap mass spectrometer (MS) (Thermo Fisher Scientific, Waltham, MA, USA). Separation was performed on a Luna C18 100 Å column (250 mm × 4.6 mm, 5 μm) with a 4 × 3 mm i.d. guard column of the same material (Phenomenex, Torrance, CA). The separation temperature was 35 °C and the flow rate was 1 mL/min at an injection volume of 20 μL. Separation took place in a gradient system using two solvents: phase A: 1% (*v*/*v*) formic acid in water and phase B: 83/20/17 (*v*/*v*/*v*) acetonitrile/methanol/water). The following gradient was used: 0−6 min 5% of phase B, 6−36 min 5−28% of phase B; 36−48 min 28−73% of phase B; 48−54 min 73% of phase B; 54−60 min 73−5% of phase B; and 60−70 min 5% of phase B. The DAD detector recorded spectra simultaneously in the range of 200–600 nm. The mass spectrometer recorded spectra in negative mode (H-ESI source). The source parameters were set as follows: vaporizer temperature 500 °C, ion spray voltage 4 kV, and capillary temperature 400 °C; with sheath gas and auxiliary gas flow rates being 75 and 20 units, respectively. In full MS/dd-MS2 scanning mode, the mass range was set at *m*/*z* 200–2000 and collision energy at 20 eV. Data were collected using Xcalibur software (Thermo Fisher Scientific, Waltham, MA, USA). The results of polyphenols identification are given in Table 1.

### 3.6. Quantitation of Polyphenols

The content of individual polyphenolic compounds was determined according to the methodology described in a previous publication [41] using a Smartline (Knauer, Berlin, Germany) chromatograph equipped with degasser unit (Manager 5000), two pumps (P1000), a mixing chamber, an autosampler (3950), a column oven, and a PDA detector (2800). Separation was performed on a Gemini C18 110 Å column (250 mm × 4.6 mm, 5 μm) with a precolumn of the same material (Phenomenex, Torrance, CA). The separation temperature was 35 °C and the flow rate was 1.25 mL/min at an injection volume of 20 μL. Separation took place in a gradient system using two solvents: phase A: 0.05% (*v*/*v*) phosphoric acid in water and phase B: 83/20/17 (*v/v/v*) acetonitrile/methanol/water). The gradient program was as follows: 0–5 min 4% of phase B; 5–12.5 min 4–15% of phase B; 12.5–42.5 min 15–40% of phase B; 42.5–51.8 min 40–50% of phase B; 51.8–53.4 min 50–55% of phase B; and 53.4–55 min 4% of phase B. Agrimoniin was detected at 250 nm, while flavonoids and ellagic acid were detected at 360 nm. Calculation of the polyphenol content was performed on the basis of calibration curves prepared for individual standards. Standard curves were used to determine the content of polyphenols in the following concentration ranges: 5.0–249 mg/L for agrimoniin (LOD = 1.29 mg/L, LOQ = 3.901 mg/L, R^2^ = 0.999), 2.5–76.6 mg/L for ellagic acid (LOD = 0.168 mg/L, LOQ = 0.508 mg/L, R^2^ = 0.999), 4.1–41.4 mg/L for quercetin 3-*O*-glucoside (LOD = 0.202 mg/L, LOQ = 0.611 mg/L, R^2^ = 0.999), 1.14–11.4 mg/L for quercetin 3-*O*-rhamnoglucoside (LOD = 0.219 mg/L, LOQ = 0.663 mg/L, R^2^ = 0.998), 1.6–16.0 mg/L for quercetin 3-*O*-galactoside (LOD = 0.013 mg/L; LOQ = 0.039 mg/L, R^2^ = 0.999), 1.4–14.0 mg/L for kaempferol 3-*O*-glucoside (LOD = 0.187 mg/L, LOQ = 0.566 mg/L, R^2^ = 0.999), 1.2–12.0 mg/L for KpCG (LOD = 0.042 mg/L, LOQ = 0.127 mg/L, R^2^ = 0.999), 1.23–12.3 mg/L for luteolin (LOD = 0.019 mg/L, LOQ = 0.058 mg/L, R^2^ = 0.998), and 1.0–100.0 mg/L for apigenin 7-*O*-glucoside (LOD = 0.148 mg/L, LOQ = 0.448 mg/L, R^2^ = 0.999). The LOD and LOQ were determined based on the standard deviation of the response (SD) and the slope of the calibration curve (S) according to the formula: LOD = 3(SD/S), LOQ = 10(SD/S) [44]. Table S3 in Supplementary Materials contains validation parameters. Agrimoniin, ellagic acid, quercetin 3-*O*-rhamnoside, quercetin 3-*O*-galactoside, kaempferol 3-*O*-glucoside, KpCG, and apigenin 7-*O*-glucoside were quantified using their respective standards. Quercetin arabinoglycoside was quantified as quercetin 3-*O*-glucoside equivalents; quercetin 3-*O*-rhamnoglucoside was quantified as quercetin 3-*O*-rhamnoside equivalents; apigenin 7-*O*-glucuronide, apigenin-8-*C*-glucoside, and apigenin-6-*C*-glucoside were quantified as apigenin 7-*O*-glucoside equivalents; and luteolin 7-*O*-glucoside and luteolin 7-*O*-glucuronide were quantified as luteolin equivalents.

Polyphenolic content in *A. procera* and *A. eupatoria* leaves and stems in particular stages of plant development is expressed as the arithmetic mean calculated on the basis of the amount of polyphenols determined in leaves and stems collected at different harvest times (BRs) within a given stage of plant development.

As regards polyphenolic content in the fruits, seeds, and hypanthia of the two studied agrimony species, the data presented for a given stage of plant development (S3, S4, S5) refer to the mean amount of polyphenols in combined samples from the respective plant parts collected at harvest times within a given stage.

All results were expressed as mg/100 g DW and presented in Table 2 and Table 3, respectively, for *A. procera* and *A. eupatoria*.

### 3.7. Statistical Analysis

The results are expressed as means and pooled SEM. One-way analysis of variance (ANOVA) and the post hoc Duncan test at a statistical significance of *p* ≤ 0.05 were used to evaluate differences in polyphenolic content between the five main stages of plant development in the various aerial parts of *A. procera* and *A. eupatoria*.

Principal component analysis (PCA) was used to explain polyphenolic profile variation between samples in a set consisting of *A. procera* and *A. eupatoria* leaves, stems, fruits, seeds, and hypanthia. The experimental layout consisted of 134 observations and 11 variables (content of individual polyphenols) for *A. procera*, and 134 observations and 12 variables for *A. eupatoria*. To provide comparable weights for all parameters, all obtained data were autoscale-preprocessed. Therefore, each variable was mean centered, and variance was scaled to unity. In the absence of compound content, a value of “0” was used in the PCA analysis. Statistical analysis was performed using Statistica version 10.0 software (StatSoft, Tulsa, OK, USA).

## 4. Conclusions

The present study investigated the qualitative and quantitative polyphenolic composition of *A. procera* and *A. eupatoria* leaves, stems, fruits, seeds, and hypanthia in the main stages of vegetative development. The findings indicate that while the two plant species differ substantially in terms of the qualitative and quantitative content of those compounds, both are potentially good sources of polyphenols. *A. procera* leaves and stems are distinguished by very high levels of agrimoniin (which is practically the only ellagitannin they contain). *A. eupatoria* exhibits a moderate agrimoniin concentration in its leaves, but it also has valuable apigenin *C*-glycosides. As fruits, seeds and hypanthia constitute only a small part of the plant, the obtained results on polyphenol composition are primarily of scientific interest, while leaves and stems remain the most important source of agrimony polyphenols. Taking into account the content of agrimoniin and glycosides of apigenin, luteolin and quercetin, the use of *A. procera* is particularly interesting.

## Figures and Tables

**Table 1:** LC-MS identification of agrimoniin and flavonoids in the leaves, stems, fruits, seeds, and hypanthia of *A. procera* and *A. eupatoria*.

Peak No.	Compound	RT [min]	UV [nm]	MS Data [*m*/*z*]	MS/MS Data	Occurrence					Identification
						*A. procera* Wallr.		*A. eupatoria* L.			
						L S F H	Se	L S	F H	Se	
1	Quercetin arabinoglycoside ^a^	35.5	257, 355	[595.14] ^−1^	463, 445, **301** *	+	+	−	−	−	[3]
2	Agrimoniin	36.3	260sh	[934.08] ^−2^	1567, 1235, 1085, 935, 897, 783, 633, **301**	+	+	+	+	+	Standard, [8,13,33]
3	Apigenin 8-*C*-glucoside	37.9	270, 353	[431.07] ^−1^	341, **311**	−	−	+	+	−	Standard, [8]
4	Quercetin 3-*O*-rhamnoglucoside	38.3	257, 354	[609.12] ^−1^	463, 343, **301**	+	−	+	+	−	Standard, [8,13,14,15,34]
5	Apigenin 6-*C*-glucoside	38.7	268, 350	[431.08] ^−1^	341, **311**	−	−	+	+	−	Standard, [8,12,13,14]
6	Ellagic acid	38.8	254, 350	[301.10] ^−1^	−	+	+	+	+	+	Standard, [8]
7	Quercetin 3-*O*-galactoside	39.4	257, 353	[463.08] ^−1^	343, **301**	+	−	+	+	−	Standard, [12,13,14]
8	Kaempferol 3-*O*-glucoside	40.0	267, 350	[447.09] ^−1^	327, **285**, 269, 255, 151	+	−	−	−	−	Standard, [12,13,15,34]
9	Luteolin 7-*O*-glucuronide ^a^	40.6	266, 349	[461.07] ^−1^	357, 327, **285**, 175, 151, 113	+	−	+	+	−	[7,8,13,34]
10	Luteolin 7-*O*-glucoside ^a^	42.5	267, 337	[447.12] ^−1^	327, **285**, 175, 151, 113	+	−	+	−	−	[7,8,34]
11	Apigenin 7-*O*-glucuronide ^a^	43.1	268, 339	[445.07] ^−1^	**269**, 175, 113	+	−	+	+	+	[7,8,13,34]
12	Apigenin 7-*O*-glucoside ^a^	43.5	269, 343	[431.08] ^−1^	311, 327, **269**, 175	+	−	+	+	−	Standard, [8,13,15]
13	Quercetin 3-*O*-rhamnoside	44.3	261, 350	[447.09] ^−1^	343, **301**, 179	−	−	+	+	−	Standard, [7,8,13,34]
14	KpCG *	46.2	269, 315	[593.12] ^−1^	447, 307, **285**	+	+	+	+	+	Standard, [12,34]
15	KpCG * isomer	46.7	269, 315	[593.12] ^−1^	447, 307, **285**	+	+	+	+	+	[12,34]

RT—retention time; ^a^—tentative assignment based on MS and UV-Vis spectra, and literature data; * MS/MS value and MS/MS data are experimental values; * MS/MS ions marked in bold font represent fragments with intensity is >50% relative to the maximum intensity; standard—identification based on the standard compound; L—leaf, S—stem, F—fruit, Se—seed, H—hypanthia; KpCG *—kaempferol-3-*O*-β-d-(6″-*E*-p-coumaroyl)-glucopyranoside (tiliroside); sh—shoulder.

**Table 2:** The content of polyphenols in the aerial parts (leaves, stems, fruits, seeds, and hypanthia) of *A. procera* at the main stages of plant development, expressed in mg/100 g of dry weight (DW) * and, in brackets, percentage of each polyphenol related to the total polyphenol content.

	Agrimoniin	Ellagic Acid	Quercetin Arabinoglycoside	Quercetin 3-*O*-rhamnoglucoside	Quercetin 3-*O*-galactoside	Kaempferol 3-*O*-glucoside	Sum of KpCG * Isomer	Luteolin 7-*O*-glucuronide	Luteolin 7-*O*-glucoside	Apigenin 7-*O*-glucuronide	Apigenin 7-*O*-glucoside	Total
Stage of Development												
LEAVES												
S1	2166.0 ± 402.0c (49.8)	39.4 ± 7.1a (0.9)	611.0 ± 27.5b (14.1)	88.5 ± 5.3c (2.0)	96.2 ± 16.3b (2.2)	84.2 ± 4.2b (1.9)	58.3 ± 5.3c (1.3)	181.2 ± 7.7c (4.0)	29.0 ± 4.1a (0.6)	1015.1 ± 8.5c (22.6)	113.4 ± 8.5b (2.5)	4482.2 ± 356.8c
S2	2002.8 ± 306.7bc (45.9)	49.7 ± 9.1c (1.1)	653.4 ± 44.9c (15.0)	83.2 ± 6.2c (1.9)	105.6 ± 13.0bc (2.4)	81.3 ± 4.7b (1.9)	53.5 ± 6.6bc (1.2)	176.3 ± 14.7c (4.0)	32.5 ± 2.3a (0.7)	1007.4 ± 90.1c (23.1)	117.3 ± 8.5b (2.7)	4362.7 ± 395.4c
S3	1872.1 ± 128.8b (49.1)	38.2 ± 2.9a (1.0)	551.5 ± 44.9a (14.5)	66.0 ± 4.3b (1.7)	81.9 ± 16.4a (2.1)	65.9 ± 4.5a (1.7)	59.6 ± 7.0c (1.6)	141.0 ± 15.4ab (3.7)	29.0 ± 5.5a (0.8)	808.4 ± 104.7a (21.2)	98.5 ± 15.9a (2.6)	3812.2 ± 143.0b
S4	1583.6 ± 176.1a (45.8)	41.4 ± 5.5ab (1.2)	569.4 ± 67.7ab (16.5)	66.4 ± 8.3b (1.9)	101.8 ± 17.5bc (2.9)	68.0 ± 9.5a (2.0)	49.7 ± 11.4b (1.4)	152.9 ± 18.5b (4.4)	37.4 ± 5.4b (1.1)	911.0 ± 101.7b (26.4)	119.1 ± 13.8b (3.4)	3455.6 ± 510.3a
S5	1595.6 ± 166.0a (44.1)	47.0 ± 7.0bc (1.3)	530.2 ± 27.2a (14.6)	58.2 ± 5.6a (1.6)	112.9 ± 12.3c (3.1)	62.6 ± 5.5a (1.7)	42.8 ± 4.2a (1.2)	138.8 ± 8.4a (3.8)	43.1 ± 3.5c (1.2)	869.3 ± 43.4ab (24.0)	120.5 ± 4.7b (3.3)	3621.0 ± 134.7ab
Mean	1855.6 ± 342.8 (46.8)	43.9 ± 8.3 (1.1)	594.7 ± 65.1 (15.0)	74.2 ± 12.6 (1.9)	100.8 ± 17.2 (2.5)	73.9 ± 10.3 (1.9)	52.8 ± 9.2 (1.3)	161.5 ± 21.8 (4.1)	34.1 ± 6.3 (0.9)	939.0 ± 112.4 (23.6)	114.9 ± 12.6 (2.9)	3963.1 ± 539.1
STEMS												
S1	2641.5 ± 457.6a (95.0)	35.1 ± 5.8ab (1.3)	61.5 ± 8.9a (2.2)	9.3 ± 1.0a (0.3)	11.0 ± 2.0a (0.4)	3.1 ± 0.5a (0.1)	8.0 ± 1.4c (0.3)	3.4 ± 0.5a (0.1)	1.9 ± 0.3a (0.1)	4.7 ± 1.2a (0.2)	1.5 ± 0.2a (0.1)	2781.1 ± 466.5a
S2	2703.7 ± 588.4a (93.7)	41.0 ± 10.8c (1.4)	86.1 ± 18.4b (3.0)	10.4 ± 1.8ab (0.4)	14.4 ± 3.3b (0.5)	4.3 ± 1.1b (0.1)	6.8 ± 0.9b (0.8)	5.2 ± 1.5b (0.2)	2.7 ± 0.5b (0.1)	9.5 ± 3.0b (0.3)	2.8 ± 0.9b (0.1)	2886.8 ± 621.8a
S3	3178.4 ± 84.3b (94.2)	31.7 ± 1.1a (0.9)	103.7 ± 7.3c (3.1)	10.8 ± 0.8b (0.3)	14.1 ± 1.2b (0.4)	4.7 ± 0.6b (0.1)	5.8 ± 0.3a (0.2)	6.1 ± 0.7bc (0.2)	3.1 ± 0.4bc (0.1)	12.0 ± 2.4bc (0.4)	3.5 ± 0.5b (0.1)	3373.9 ± 87.3b
S4	3166.4 ± 493.7b (93.6)	31.7 ± 5.9a (0.9)	114.9 ± 27.5c (3.4)	11.8 ± 2.3b (0.4)	16.4 ± 3.8bc (0.5)	5.1 ± 1.4bc (0.2)	5.5 ± 1.0a (0.2)	7.2 ± 2.0cd (0.2)	3.6 ± 1.2c (0.1)	16.9 ± 5.1d (0.5)	4.3 ± 1.2bc (0.1)	3383.8 ± 540.0b
S5	3926.7 ± 420.7c (94.0)	36.6 ± 2.6ab (0.9)	141.8 ± 10.5d (3.4)	13.3 ± 0.8c (0.3)	17.6 ± 1.7c (0.4)	5.8 ± 0.5c (0.1)	5.1 ± 0.3a (0.1)	7.7 ± 0.4d (0.2)	4.3 ± 0.5d (0.1)	14.6 ± 1.2cd (0.3)	4.5 ± 0.5c (0.1)	4178.1 ± 437.6c
Mean	3038.8 ± 628.5 (94.0)	35.9 ± 8.0 (1.1)	98.9 ± 30.4 (3.1)	11.0 ± 2.0 (0.3)	14.7 ± 3.5 (0.5)	4.5 ± 1.3 (0.1)	6.3 ± 1.3 (0.4)	5.9 ± 1.9 (0.2)	3.1 ± 1.0 (0.1)	11.5 ± 5.2 (0.4)	3.1 ± 1.3 (0.1)	3233.8 ± 669.4
FRUITS												
S3	2082.1 ± 51.9b (59.1)	161.8 ± 6.3b (4.6)	672.5 ± 0.4b (19.1)	74.8 ± 0.9c (2.1)	141.2 ± 1.0c (4.0)	25.5 ± 0.1c (0.7)	38.4 ± 0.6b (1.1)	83.9 ± 0.1b (2.4)	15.5 ± 1.4a (0.4)	108.1 ± 2.0b (3.1)	12.5 ± 1.2b (0.4)	3526.0 ± 71.8b
S4	1533.4 ± 83.8a (56.2)	127.3 ± 7.3a (4.7)	569.8 ± 43.6a ()20.9	64.2 ± 4.2b (2.4)	100.1 ± 7.0b (3.7)	19.1 ± 1.1b (0.7)	23.9 ± 1.8a (0.9)	74.1 ± 5.9b (2.7)	13.4 ± 1.0a (0.5)	102.4 ± 8.0b (3.8)	6.3 ± 0.3a (0.2)	2728.9 ± 170.8a
S5	1414.1 ± 17.0a ()57.3	127.4 ± 2.8a (5.2)	498.0 ± 1.0a (20.2)	51.4 ± 1.6a (2.1)	77.9 ± 0.8a (3.2)	14.5 ± 0.3a (0.6)	35.0 ± 0.1b (1.4)	61.7 ± 0.3a (2.5)	12.9 ± 0.1a (0.5)	83.9 ± 1.3a (3.4)	5.3 ± 0.5a (0.2)	2469.4 ± 19.0a
Mean	1676.5 ± 321.8 (57.7)	138.8 ± 18.3 (4.8)	580.1 ± 80.9 (19.9)	63.5 ± 10.6 (2.2)	106.4 ± 28.9 (3.7)	19.7 ± 5.0 (0.7)	32.4 ± 6.9 (1.1)	73.2 ± 10.3 (2.5)	13.9 ± 1.5 (0.5)	98.1 ± 11.9 (3.4)	8.0 ± 3.5 (0.3)	2908.1 ± 499.5
SEEDS												
S3	2036.3 ± 71.7c (93.3)	139.8 ± 5.2b (6.4)	2.0 ± 0.2a (0.1)	nd	nd	nd	4.8 ± 0.3b (0.2)	nd	nd	nd	nd	2183.9 ± 67.4c
S4	1754.9 ± 59.4b (92.8)	129.7 ± 8.7ab (6.9)	2.3 ± 0.1a (0.1)	nd	nd	nd	4.4 ± 0.5b (0.2)	nd	nd	nd	nd	1891.3 ± 68.5b
S5	1189.0 ± 25.2a (87.9)	115.1 ± 0.9a (8.5)	8.3 ± 0.5b (0.6)	nd	nd	nd	1.9 ± 0.1a (0.3)	nd	nd	nd	nd	1353.9 ± 24.6a
Mean	1660.1 ± 388.4 (91.8)	128.2 ± 12.0 (7.1)	4.2 ± 3.2 (0.2)	nd	nd	nd	4.6 ± 0.4 (0.3)	nd	nd	nd	nd	1809.1 ± 379.2
HYPANTHIA												
S3	236.2 ± 13.3a (26.8)	37.5 ± 1.9a (4.3)	383.2 ± 2.9a (43.4)	38.9 ± 0.2a (4.4)	59.5 ± 0.3ab (6.7)	9.7 ± 0.3a (1.1)	8.8 ± 0.3a (1.0)	44.2 ± 0.2a (5.0)	7.2 ± 0.4a (0.9)	52.1 ± 1.6a (5.9)	4.7 ± 1.3a (0.5)	882.2 ± 17.5a
S4	255.3 ± 57.0a (29.2)	38.8 ± 7.5a (4.4)	359.1 ± 94.3a (41.1)	39.7 ± 10.3a (4.5)	53.1 ± 12.8a (6.1)	8.7 ± 1.8a (1.0)	8.4 ± 2.3a (1.0)	45.8 ± 10.4a (5.2)	7.2 ± 1.8a (0.8)	54.2 ± 13.8a (6.2)	3.6 ± 0.3a (0.4)	873.8 ± 211.7a
S5	525.6 ± 69.1b (33.5)	110.6 ± 10.2b (7.0)	572.4 ± 38.6b (36.4)	60.8 ± 1.3b (3.9)	78.6 ± 1.2b (5.0)	13.5 ± 0.4b (0.9)	32.6 ± 2.7b (2.1)	72.3 ± 2.0b (4.6)	17.4 ± 0.6b (1.1)	78.1 ± 4.2a (5.0)	9.1 ± 0.2b (0.6)	1571.1 ± 130.2b
Mean	339.0 ± 150.3 (30.6)	62.3 ± 37.8 (5.6)	438.2 ± 114.0 (39.5)	46.5 ± 12.1 (4.2)	63.7 ± 13.2 (5.7)	10.7 ± 2.4 (1.0)	16.6 ± 12.5 (1.5)	54.1 ± 14.9 (4.9)	10.6 ± 5.3 (1.0)	61.5 ± 14.5 (5.5)	5.8 ± 2.7 (0.5)	1109.1 ± 374.9

* The values are presented as the mean ± standard deviation in mg/100 g DW; the values in brackets refer to the share of individual polyphenols in the total polyphenols content and are given as a percentage; abc—the results in individual columns relating to the content of polyphenols in the leaves, stems, fruits, seeds and hypanthia of *A. procera* separately marked by the same roman letter do not differ statistically at *p* < 0.05; nd—not detected; S—stage of development; S1—the development of vegetative parts and the appearance of inflorescence; S2—the flowering stage; S3—the fruit and seed development stage; S4—the beginning of fruit and seed ripening stage; S5—the fruit and seed full maturity stage; KpCG *—kaempferol-3-*O*-β-d-(6″-*E*-p-coumaroyl)-glucopyranoside (tiliroside).

**Table 3:** The content of polyphenols in the aerial parts (leaves, stems, fruits, seeds, and hypanthia) of *A. eupatoria* at the main stages of plant development, expressed in mg/100 g of dry weight (DW) * and, in brackets, the percentage of each polyphenol related to the total polyphenol content.

	Agrimoniin	Ellagic Acid	Quercetin 3-*O*-rhamnoglucoside	Quercetin 3-*O*-galactoside	Quercetin 3-*O*-rhamnoside	Sum of KpCG*Isomer	Luteolin 7-*O*-glucuronide	Luteolin 7-*O*-glucoside	Apigenin 7-*O*-glucuronide	Apigenin 7-*O*-glucoside	Apigenin 8-*C*-glucoside	Apigenin 6-*C*-glucoside	Total
Stage of Development													
LEAVES													
S1	417.3 ± 45.5a (30.0)	12.9 ± 3.4d (0.9)	23.0 ± 4.5a (1.7)	85.1 ± 18.5a (10.7)	102.9 ± 17.2b (7.4)	47.5 ± 8.7c (3.4)	145.4 ± 23.8b (10.5)	11.7 ± 2.2a (0.8)	222.8 ± 25.8b (16.0)	55.8 ± 7.6ab (4.0)	53.0 ± 6.5b (3.8)	149.9 ± 14.9ab (10.8)	1327.4 ± 109.6a
S2	419.9 ± 71.4a (31.5)	9.6 ± 3.5c (0.7)	28.6 ± 6.4ab (2.2)	98.6 ± 22.4a (7.4)	103.1 ± 13.5b (7.7)	51.4 ± 9.6c (3.9)	130.2 ± 18.3b (9.8)	13.8 ± 2.8ab (1.0)	221.0 ± 18.8b (16.6)	56.3 ± 6.5ab (4.2)	53.8 ± 4.1b (4.0)	144.7 ± 15.3ab (10.9)	1331.0 ± 80.1a
S3	491.2 ± 65.4c (39.0)	6.1 ± 2.1ab (0.5)	22.6 ± 3.3a (1.8)	81.5 ± 7.3a (6.5)	87.1 ± 12.6a (6.9)	35.2 ± 4.1ab (2.8)	98.8 ± 14.6a (7.8)	12.6 ± 1.9a (1.0)	185.5 ± 22.1a (14.7)	52.7 ± 6.4a (4.2)	49.9 ± 1.7b (4.0)	137.3 ± 11.6a (10.9)	1260.7 ± 60.9a
S4	420.0 ± 70.9a (33.2)	7.5 ± 2.8bc (0.6)	34.0 ± 16.3b (2.7)	98.9 ± 39.0a (7.8)	90.7 ± 18.2ab (7.2)	32.5 ± 5.3a (2.6)	104.3 ± 18.6a (8.2)	18.3 ± 10.1b (1.4)	188.7 ± 33.5a (14.9)	64.2 ± 16.9bc (5.1)	41.2 ± 7.7a (3.3)	165.2 ± 47.6b (13.0)	1265.6 ± 146.3a
S5	401.0 ± 43.1a (30.1)	4.6 ± 0.6a (0.3)	47.1 ± 5.6c (3.5)	126.8 ± 18.9b (9.5)	103.1 ± 6.9a (7.7)	40.2 ± 4.6b (3.0)	112.8 ± 6.0a (8.5)	25.7 ± 4.5c (1.9)	202.8 ± 13.6ab (15.2)	70.4 ± 9.1c (5.3)	50.8 ± 4.6b (3.8)	146.7 ± 25.5ab (11.0)	1332.0 ± 91.7a
Mean	426.7 ± 666.8 (32.4)	8.5 ± 3.9 (0.6)	30.7 ± 11.9 (2.3)	98.3 ± 24.0 (8.3)	97.9 ± 15.7 (7.4)	42.4 ± 10.6 (3.2)	119.9 ± 24.0 (9.1)	16.0 ± 7.1 (1.2)	206.2 ± 28.3 (15.7)	59.6 ± 11.7 (4.5)	49.7 ± 7.3 (3.8)	149.8 ± 28.4 (11.4)	1305.9 ± 130.6
STEMS													
S1	175.5 ± 31.8a (74.5)	16.4 ± 5.3cd (6.9)	2.9 ± 1.1a (1.2)	5.3 ± 1.52a (2.2)	5.1 ± 1.2a (2.2)	10.6 ± 2.2c (4.5)	3.2 ± 0.8a (1.4)	0.9 ± 0.3a (0.4)	2.1 ± 0.5a (0.9)	1.1 ± 0.2a (0.5)	0.7 ± 0.2a (0.3)	11.7 ± 3.6bc (5.0)	235.6 ± 35.9a
S2	161.4 ± 24.9a (68.1)	17.8 ± 3.7d (7.5)	5.9 ± 1.7b (2.5)	10.0 ± 3.0ab (4.2)	7.1 ± 2.2ab (3.0)	9.3 ± 2.2bc (3.9)	5.0 ± 1.3b (2.1)	1.4 ± 0.3b (0.6)	3.0 ± 0.5b (1.3)	1.7 ± 0.6b (0.7)	1.2 ± 0.2b (0.5)	13.2 ± 2.4c (5.6)	237.1 ± 29.5a
S3	246.2 ± 37.3b (76.6)	14.3 ± 1.4c (4.5)	5.9 ± 2.2b (1.8)	11.6 ± 4.3b (3.6)	8.6 ± 2.5b (2.7)	8.7 ± 1.2ab (2.7)	5.6 ± 2.0bc (1.8)	1.4 ± 0.3b (0.4)	3.9 ± 0.4c (1.2)	1.8 ± 0.3b (0.6)	1.7 ± 0.2c (0.5)	11.7 ± 1.1bc (3.6)	321.4 ± 46.1b
S4	330.4 ± 101.9c (80.9)	10.6 ± 2.9b (2.6)	7.7 ± 3.3b (1.9)	17.7 ± 9.5c (4.3)	8.3 ± 2.9b (2.0)	7.1 ± 1.8a (1.7)	6.9 ± 3.1cd (1.7)	1.6 ± 0.6bc (0.4)	4.1 ± 0.5c (1.0)	2.0 ± 0.9b (0.5)	2.6 ± 0.5e (0.6)	9.7 ± 2.1ab (2.4)	408.7 ± 102.8c
S5	236.9 ± 35.1b (72.5)	6.7 ± 1.9a (2.0)	12.3 ± 4.4c (3.8)	22.7 ± 6.4c (7.0)	11.4 ± 2.0c (3.5)	8.8 ± 1.6ab (2.7)	8.6 ± 2.2d (2.6)	1.8 ± 0.6c (0.6)	4.2 ± 0.3c (1.3)	3.1 ± 0.7c (0.9)	2.2 ± 0.1d (0.7)	8.0 ± 4.1a (2.4)	326.7 ± 39.9b
Mean	226.8 ± 91.2 (75.0)	13.8 ± 5.2 (4.6)	6.7 ± 3.7 (2.2)	13.0 ± 7.9 (4.3)	7.8 ± 2.9 (3.0)	8.9 ± 2.2 (2.9)	5.7 ± 2.6 (1.9)	1.4 ± 0.5 (0.5)	3.4 ± 0.9 (1.1)	1.9 ± 0.8 (0.6)	1.7 ± 0.8 (0.6)	11.2 ± 3.3 (3.7)	302.3 ± 95.2
FRUITS													
S3	1004.7 ± 80.3c (77.4)	49.9 ± 5.4a (3.8)	6.7 ± 0.3c (0.5)	28.6 ± 0.1c (2.2)	7.9 ± 0.6c (0.6)	20.3 ± 0.1b (1.6)	4.2 ± 0.4b (0.3)	nd	77.3 ± 3.0b (5.9)	6.0 ± 0.1c (0.5)	8.3 ± 0.1b (0.6)	63.7 ± 0.9c (4.9)	1297.7 ± 84.8b
S4	639.6 ± 10.9b (75.8)	46.3 ± 0.4a (5.5)	4.3 ± 0.2a (0.5)	17.6 ± 0.1a (2.1)	4.7 ± 0.2a (0.6)	13.8 ± 3.2a (1.6)	2.9 ± 0.1a (0.3)	nd	59.4 ± 1.6a (7.0)	2.9 ± 0.1a (0.3)	7.4 ± 0.1a (0.9)	39.4 ± 0.5a (4.7)	843.5 ± 17.2a
S5	470.3 ± 52.2a (67.3)	49.6 ± 1.7a (7.5)	5.1 ± 0.1b (0.7)	25.5 ± 0.5b (3.7)	6.7 ± 0.2b (1.0)	13.6 ± 0.1a (2.0)	3.2 ± 0.0a (0.5)	nd	62.6 ± 2.2a (9.0)	3.7 ± 0.2b (0.5)	7.6 ± 0.1a (1.1)	47.7 ± 0.5b (6.8)	698.4 ± 54.5a
Mean	704.8 ± 248.0 (74.5)	48.6 ± 3.1 (5.1)	5.4 ± 1.1 (0.6)	23.9 ± 5.1 (2.5)	6.4 ± 1.5 (0.7)	15.9 ± 3.7 (1.7)	3.4 ± 0.6 (0.4)	nd	66.3 ± 8.6 (7.0)	4.2 ± 1.4 (0.4)	7.7 ± 0.4 (0.8)	50.3 ± 11.1 (5.3)	946.5 ± 283.3
SEEDS													
S3	720.5 ± 37.0c (92.6)	52.3 ± 2.8ab (6.7)	nd	nd	nd	2.6 ± 0.3a (0.3)	nd	nd	3.1 ± 0.1b (0.4)	nd	nd	nd	778.5 ± 40.4c
S4	549.9 ± 21.1b (89.8)	59.9 ± 5.7b (9.8)	nd	nd	nd	2.4 ± 0.6a (0.4)	nd	nd	2.4 ± 0.2a (0.4)	nd	nd	nd	614.7 ± 27.6b
S5	307.3 ± 68.7a (85.8)	46.9 ± 0.5a (13.1)	nd	nd	nd	1.6 ± 0.4a (0.5)	nd	nd	2.4 ± 0.3a (0.7)	nd	nd	nd	358.2 ± 68.5a
Mean	529.9 ± 189.2 (90.1)	53.0 ± 6.5 (9.1)	nd	nd	nd	2.2 ± 0.6 (0.4)	nd	nd	2.6 ± 0.4 (0.4)	nd	nd	nd	583.8 ± 193.2
HYPANTHIA													
S3	164.4 ± 18.8a (47.8)	21.7 ± 0.9a (6.3)	4.5 ± 0.4a (1.3)	16.6 ± 2.8a (4.8)	4.0 ± 0.4a (1.2)	8.5 ± 0.6a (2.5)	2.9 ± 0.1b (0.9)	nd	70.8 ± 9.3a (20.6)	3.1 ± 0.3a (0.9)	6.8 ± 0.8a (2.0)	40.4 ± 6.2a (11.8)	343.9 ± 40.4a
S4	285.7 ± 52.4ab (50.3)	33.1 ± 5.4a (5.8)	10.9 ± 2.1b (1.9)	32.6 ± 6.3a (5.7)	8.1 ± 1.2a (1.4)	9.7 ± 1.5ab (1.7)	5.8 ± 1.1c (1.0)	nd	100.5 ± 16.5a (17.7)	8.7 ± 1.3b (1.5)	9.9 ± 1.8a (1.7)	63.2 ± 12.4a (11.1)	568.3 ± 102.1ab
S5	383.2 ± 55.7b (52.6)	76.0 ± 12.0b (10.4)	11.7 ± 3.1b (1.6)	41.2 ± 12.9a (5.7)	18.1 ± 4.3b (2.5)	16.6 ± 3.4b (2.3)	2.1 ± 0.1a (0.3)	nd	92.9 ± 25.1a (12.7)	7.8 ± 1.4b (1.1)	11.5 ± 2.7a (1.6)	69.7 ± 22.8a (9.6)	728.6 ± 143.7b
Mean	277.8 ± 104.2 (50.8)	46.3 ± 26.3 (8.0)	9.0 ± 3.9 (1.7)	30.1 ± 13.0 (5.5)	10.1 ± 6.8 (1.8)	11.6 ± 4.3 (2.1)	3.6 ± 2.1 (0.6)	nd	88.1 ± 19.7 (16.1)	6.6 ± 2.8 (1.2)	9.4 ± 2.6 (1.7)	57.8 ± 18.2 (10.6)	546.9 ± 190.9

* The values are presented as the mean ± standard deviation in mg/100 g DW; the values in brackets refer to the share of individual polyphenols in the total polyphenols content and are given as a percentage; abc—the results in the individual columns relating to the content of polyphenols in the leaves, stems, fruits, seeds and hypanthia of *A. eupatoria* separately marked by the same roman letter do not differ statistically at *p* < 0.05; nd—not detected; S—stage of development; S1—the development of vegetative parts and the appearance of inflorescence; S2—the flowering stage; S3—the fruit and seed development stage; S4—the beginning of fruit and seed ripening stage; S5—the fruit and seed full maturity stage; KpCG*—kaempferol-3-*O*-β-d-(6″-*E*-p-coumaroyl)-glucopyranoside (tiliroside).

## Data Availability

The data presented in this study are available on request from the corresponding author.

## Supplementary Materials

The following are available online, Figure S1: UHPLC-ESI-MS chromatograms of *Agrimonia procera* Wallr. and *Agrimonia eupatoria* L. leaves extract, Table S1: Eigenvalues and eigenvectors from PCA analysis for *Agrimonia procera* Wallr., Table S2: Eigenvalues and eigenvectors from PCA analysis for *Agrimonia eupatoria* L., Table S3: Analytical parameters used for quantitative analysis.

## Sample Availability

Samples of the compounds are not available from the authors.
