# Supplementary material for: The Aerial Parts of Agrimonia procera Wallr. and Agrimonia eupatoria L. as a Source of Polyphenols, and Especially Agrimoniin and Flavonoids
Source: Molecules. 2021 Dec 20;26(24):7706. doi: 10.3390/molecules26247706 (PMC8705483; doi:10.3390/molecules26247706)
Supplement: Supplementary file 1 [file molecules-26-07706-s001.zip › molecules-1491882-supplementary.pdf]

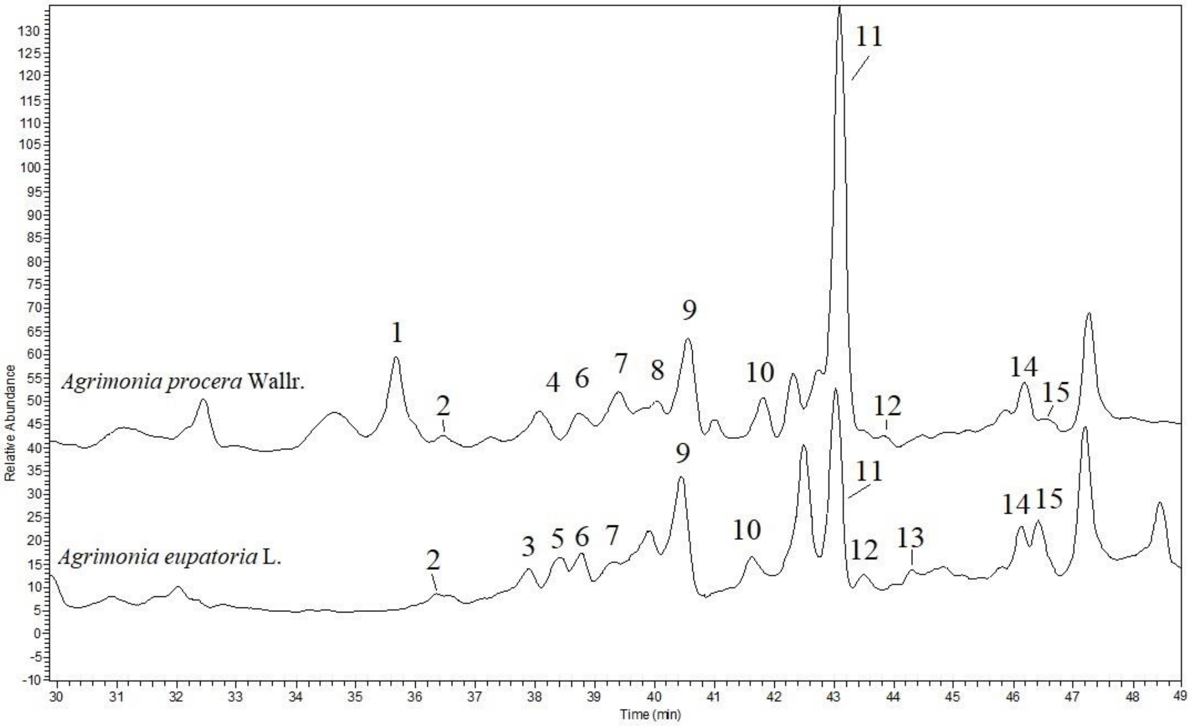

Figure S1. UHPLC-ESI-MS chromatograms of *Agrimonia procera* Wallr. and *Agrimonia eupatoria* L. leaves extract. Peak numbers correspond to Table 1.

Table S1. Eigenvalues and eigenvectors from PCA analysis for *Agrimonia procera* Wallr.

	PC1	PC2	PC3	PC4	PC5	PC6
**Eigenvalues**	8.73	1.30	0.54	0.18	0.16	0.06
Proportion of total variance	79.39	11.86	4.91	1.64	1.43	0.56
Cumulative proportion of total variance	79.39	91.25	96.16	97.81	99.24	99.80
**Eigenvectors**						

Compound	PC1	PC2
Agrimoniin	0.549058	0.558452
Ellagic acid	-0.015257	-0.923788
Quercetin arabinoglycoside	-0.966533	-0.118485
Quercetin 3-O-rhamnoglucoside	-0.970366	-0.058606
Quercetin 3-O-galactoside	-0.949280	-0.155712
Keampferol 3-O glucoside	-0.977404	0.155143
Sum of kaempferol-3-O-β-d-(6″-E-pcoumaroyl)-glucopyranoside isomer	-0.956959	0.024811
Luteolin 7-O-glucuronide	-0.993645	0.053699
Luteolin 7-O-glucoside	-0.960692	0.061087
Apigenin 7-O-glucuronide	-0.971336	0.177226
Apigenin 7-O-glucoside	-0.964197	0.187047

Table S2. Eigenvalues and eigenvectors from PCA analysis for *Agrimonia eupatoria* L.

	PC1	PC2	PC3	PC4	PC5	PC6
**Eigenvalues**	9.09	1.41	0.51	0.38	0.34	0.11
Proportion of total variance	75.78	11.77	4.27	3.19	2.83	0.89
Cumulative proportion of total variance	75.78	87.55	91.82	95.01	97.84	98.73
**Eigenvectors**						

Compound	PC1	PC2
Agrimoniin	-0.404565	-0.785010
Ellagic acid	0.461876	-0.766948
Quercetin 3-O-rhamnoglucoside	-0.904345	0.157717

Quercetin 3-O-galactoside	-0.828027	-0.027387
Quercetin 3-O-rhamnoside	-0.989227	0.033654
Sum of kaempferol-3-O-β-d-(6″-E-pcoumaroyl)-glucopyranoside isomer	-0.928683	-0.095744
Luteolin 7-O-glucuronide	-0.976022	-0.053351
Luteolin 7-O-glucoside	-0.818659	0.348285
Apigenin 7-O-glucuronide	-0.974242	-0.142686
Apigenin 7-O-glucoside	-0.981983	0.021586
Apigenin 8-C-glucoside (vitexin)	-0.975031	-0.087596
Apigenin 6-C-glucoside (isovitexin)	-0.940611	-0.139067

Table S3. Analytical parameters used for quantitative analysis

	**Linear range**	**Calibration curves**	**R^2**	**LOD**	**LOQ**
Substance	mg/L			mg/L	mg/L
Agrimoniin	5.0 – 249.0	y = 18.190x – 4.580	0.9998	1.290	3.901
Ellagic acid	2.5 – 76.6	y = 16.736x – 25.347	0.9997	0.168	0.508
Quercetin 3-O-glucoside	4.1– 41.4	y = 19.976x – 39.975	0.9999	0.202	0.611
Quercetin 3-O-rhamnoglucoside	1.14 –11.4	y = 25.741x – 11.244	0.9997	0.219	0.663
Quercetin 3-O-galactoside	1.6 – 16.0	y = 20.33x – 18.862	0.9999	0.013	0.039
Kaempferol 3-O-glucoside	1.4 – 14.0	y = 16.234x – 11.244	0.9999	0.187	0.566
KpCG*	1.2 – 12.0	y = 10.282x – 5.400	0.9999	0.0419	0.127
Luteolin	1.23 – 12.30	y = 30.717x – 22.356	0.9999	0.019	0.058
Apigenin 7-O-glucoside	1.0 – 100.0	y = 17.314x – 26.702	0.9993	0.148	0.448

KpCG* - kaempferol-3-O-β-d-(6″-E-pcoumaroyl)-glucopyranoside (tiliroside).
